# Water incident related hospital activity across England between 1997/8 and 2003/4: a retrospective descriptive study

**DOI:** 10.1186/1471-2458-6-210

**Published:** 2006-08-16

**Authors:** Holly Henderson, Richard C Wilson

**Affiliations:** 1 Research Consultant, Gateshill, Middle Road, Lychett Maltravers, Poole, Dorset, BH16 6HJ, UK; 2 Senior Public Health Information Specialist, South Birmingham Primary Care Trust, Moseley Hall Hospital, Alcester Road, Birmingham, B13 8JL, UK

## Abstract

**Background:**

No one has ever reported or investigated the number of people who have been admitted to hospital for a water related incident. The purpose of this paper is to examine, the hospital activity resulting from such incidents including to length of stay, gender, age and cause.

**Methods:**

The data was extracted from the Hospital Episode Statistics (HES) for episodes with a mention of ICD 10 (V90–94, W15–16, W65–74, X38, X92, Y21) for the years 1997/8 to 2003/4. Population based rates and relative risk were calculated using the most recent Census data for England (2001).

**Results:**

The 6,793 episodes resulted in a total of 32,520 bed days with an average of length of stay of 5.0 days. Males made up 73.7% (n = 5,006) of episodes and females 26.1% (n = 1,787). Annual trends peaked in 1999–2000 at a rate of 2.4 per 100,000 and have fluctuated on alternate years there after. In terms of relative risk males are at a 2.3 to 3.0 increased annual risk of being admitted compared to females, relating to a water event. The highest annual rates were observed within the 0 – 14 age group, ranging from 3.1 to 4.2 episodes per 100,000.

**Conclusion:**

Based on these findings, for every one drowning that occurs per year there are three hospital episodes. Each of the age groups identified within the study reported an increase in hospital episodes between 2002 – 2003 and 2003 – 2004, when considering the fatality information available it would appear that although fatalities are decreasing in the similar time period, hospital episodes are increasing.

For the 0–14 age group, the cause of the injury had changed over the years, moving away from bath tub and swimming pool, to watercraft incidents (V91 – 93). For the 15 – 59 age group there had been a decline in the frequency of watercraft and water transport episodes, however, an increase in diving and jumping injury and incidents. In the over 60 age group water transport episodes remained the most frequent, with swimming pool related episodes declining and other specified drowning and submersion increasing.

More work needs to be undertaken in regard to who is admitted to hospital, when where, and how to fill gaps in knowledge and highlight information that is critical to prevention strategies.

## Background

Every year in the United Kingdom, 10,000 people will die from accidental injury and the treatment of these injuries will cost the NHS £2 billion and the consequences of injuries received at home cost society a further £25 billion [1]. Non-fatal injuries result in 720,000 people being admitted to hospital a year and more than six million visits to accident and emergency departments each year [2]. Drowning is the second leading cause of unintentional injury mortality globally behind road traffic injuries. It is estimated that a total of 409, 272 people drown each year [3]. This equates to a global incident rate of 7.4 deaths per 100, 000 people worldwide and relates to a further 1.3 million Disability Adjusted Life Years (DALYs) which are lost as a result of premature death or disability [4].

'Death' represents only the tip of the injury "iceberg" [5]. For every life lost from an injury, many more people are admitted to hospital, attend accident and emergency departments or general practitioners, are rescued by search and rescue organisations or resolve the situation themselves. It is estimated that 1.3 million people are injured as a result of near drowning episodes globally and that many more hundreds of thousands of people are affected through incidents and near misses but there are no accurate data [4].

The United Kingdom has reported a variable drowning fatality rate, the injury chart book reports a rate of 1.0 – 1.5 per 100,000 [6] and other studies suggest a rate as low as 0.5 per 100, 000 population [7] for accidental drowning and submersion, based on the International Classification of Disease 10 code W65 – 74, however, the problem is even greater and these Global Burden of Disease (GDB) figures are an underestimate of all drowning deaths, since they exclude drownings due to cataclysms (floods), water related transport accidents, assaults and suicide [3]. A recent study in Scotland highlighted this underestimation in drowning fatality data and found that the overall death rate due to drownings in Scotland 3.26 per 100,000 [8]. Even though drowning fatality rates in the United Kingdom vary, little is known about the people who are admitted to hospital after an incident either in or on water. This paper seeks to address this gap in our knowledge through the investigation of the data available on those admitted to NHS hospitals in England.

## Methods

The data was extracted from the Hospital Episode Statistics (HES). This is the national statistical database for England of the care provided by the NHS hospitals. The HES database is a record level database of inpatient hospital activity and is currently populated by taking an annual snapshot of a sub-set of the data submitted by NHS Trusts to the NHS-Wide Clearing Service (NWCS) [9]. The dataset was extracted for the financial years between 1997/8 to 2003/2004, and included information regarding gender, age, cause of admission, and length of stay. Episodes were identified as the first episode in a spell and where there was a mention of one of the following codes. Population based rates and relative risk were calculated using the most recent Census data for England (2001). Significance for both relative risk and population rates were calculated using 95% confidence intervals. Directly age standardised rates were calculated to the European standard population.

The ICD 10 codes used are;

- V90 Accident to watercraft causing drowning and submersion;

- V91 Accident to watercraft causing other injury;

- V92 Water-transport-related drowning /submersion without accident to watercraft;

- V93 Accident on board watercraft without accident to watercraft not causing drowning or submersion;

- V94 Other and unspecified water transport accidents;

- W15 Fall from a cliff;

- W16 Diving/jumping into water cause injury other than drowning or submersion;

- W65 Drowning and submersion while in bath-tub;

- W66 Drowning and submersion following fall into bath-tub;

- W67 Drowning and submersion while in swimming-pool;

- W68 Drowning and submersion following fall into swimming-pool;

- W69 Drowning and submersion while in natural water;

- W70 Drowning and submersion following fall into natural water;

- W73 Other specified drowning and submersion;

- W74 Unspecified drowning and submersion;

- X38 Victim of flood;

- X92 Assault by drowning and submersion

- Y21 Drowning and submersion undetermined intent.

## Results

### Episodes

There were 6,793 episodes resulting from water related incidents in England during this period. Diving or jumping into water causing injury other than drowning and submersion (W16), produced the highest frequency (18.5%, n = 1,256). The least frequent cause was Accident to watercraft causing drowning and submersion (V90) with only 0.7% (n = 56) (Table 1).

### Bed days

Those involved in water related incidents spent a total of 32,520 bed days (Table 1). This equated to an average length of stay of 5.0 days. The longest length of stay was for Drowning and submersion undetermined intent (Y21), 11.9 days, followed by Victim of a flood (X 38), 7.6 days. The shortest length of stay was Accident to watercraft causing drowning and submersion (V90), at 1.9 days.

### By gender

**Figure 1:**
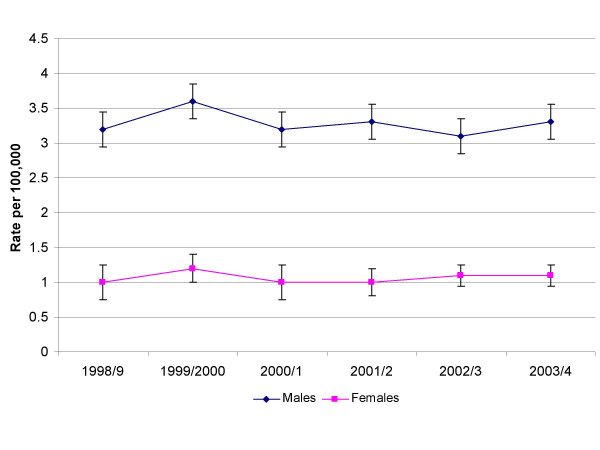
Episode rate by gender per 100,000 population, 1997/8 to 2003/4

73.7% (n = 5,006) were male and 26.3% (n = 1,787) were female. Annual trends show that both male and female episodes were highest in 1999/2000 and have dropped since, although there is no consistent trend detectable (Figure 1). This translates to a 2.3 to 3.0 significantly higher annual relative risk for males, compared to females. The annual overall water related hospital episode rate ranged from 2.0 to 2.4 per 100, 000 population in England (Table 2).

For all but four codes male was the dominant gender making up more than 70% of the episodes. The ones where the male dominance was not as great were Drowning and submersion while in bath-tub (W65), Drowning and submersion following fall into bath-tub (W66), Victim of flood (X38) and Assault by drowning and submersion (X92)(Table 3).

### By age

The 0 – 14 age group have a significantly higher rate than all other age groups with an annual rate ranging between 3.1 – 4.2 episodes per 100,000 population (Table 2).

### Over time

There are no consistent trends to be observed in the frequency of episodes by cause across the six years being studied with the numbers fluctuating annually.

### By age and ICD classification

In the 0 – 14 age group, the highest count of episodes was Unspecified Drowning and Submersion (W74) (19.7%, n = 402) (Table 4). The least frequent was Accident to water craft causing drowning and submersion (V90) (0.3%, n = 7). Compared to the other age groups they had significantly higher levels of Drowning and submersion while in a bath-tub (W65) (0.38 per 100, 000 (0.25 to 0.50)), Drowning and submersion while in a swimming pool (W67) (0.4 per 100, 000 (0.27 – 0.53), Drowning and submersion following a fall into a swimming pool (W68) (0.19 per 100, 000 (0.10 – 0.28), Other specified drowning and submersion (W73) (0.21 per 100, 000 (0.12 – 0.31)) and Unspecified drowning and submersion (W74) (0.72 per 100, 000 (0.55 – 0.90) classification. They had a significantly lower rates for Accident on board water craft without causing accident to water craft not causing drowning and submersion (V93) (0.08 per 100, 000 (0.02 – 0.14)) and Other and unspecified water transport accidents (V94) (0.05 per 100, 000 (0.0 – 0.09)).

For the 15 – 59 age group the highest frequency was for Diving and Jumping into water causing injury other than drowning and submersion (W16) (21.5 %, n = 844). The least frequent was Drowning and Submersion following a fall into a bath tub (W66) (0.3%, n = 12). Accident on board water craft without accident to watercraft not causing drowning or submersion (V93) (0.46 per 100,000 (0.38 – 0.54)), Other Unspecified water transport accidents (V94) (0.26 per 100, 000 (0.2 – 0.32) and Fall from cliff (W15) (0.35 per 100, 000 (0.28 -0.42)) were significantly higher.

In the over 60 age group the highest count was for an Accident on board water craft without accident to watercraft not causing drowning or submersion (V93) (n = 233). The least frequent hospital was for Drowning and Submersion following a fall into a swimming pool (W68) (n = 8). This age group also reported a significantly lower rates for Fall from Cliff (W15) (0.10 per 100, 000 (0.04 – 0.16)) and Diving/jumping into water causing injury other than drowning or submersion (W16) (0.13 per 100, 000 (0.06 – 0.02)).

When looking at the age standardised results the highest rates were observed for Diving/jumping into water causing injury other than drowning or submersion (W16) (0.45 per 100, 000 (0.39 – 0.51)) and Accident on board water craft without accident to watercraft not causing drowning or submersion (V93) (0.37 per 100,000 (0.31 – 0.42)).

## Discussions and conclusions

In the six years from 1997/8 to 2003/4 there was a total of 6,739 hospital episodes after a water related event. This figure has never historically been reported nor has hospital inpatient activity been related to drowning fatalities. Current figures from the Office of National Statistics show that the Number of deaths from drowning and submersion (ICD-10 W65–W74, X71, X92, Y21), England and Wales, 2001–2003 show that fatalities have declined in 2003 (2001 n = 452, 2002 n = 457, 2003 n = 437)[10]. Based on these findings, for every one drowning that occurs per year there are three hospital episodes. Using estimates from the NHS Reference costs it is estimated that these cost the NHS £2.7 M every year in emergency treatment without the additional costs of restorative surgery [17].

Water related hospital episodes as with drowning rates vary with age and gender [3,4,11,12]. They are a male phenomenon. This has already been observed within drowning and near drowning literature that incidents occurs more often in males, as it is the case for almost all types of unintentional injuries in all WHO regions [4,11] In fact the male rate of drowning is more than twice that of females and males are more likely to be hospitalised or suffer a near drowning experience [3,4]. This study reports that males have a 2.3 to 3.0 increased risk of ending up in hospital compared to females and produce a population rate that is three time higher than females. There is in general a need for realistic exposure data to inform risk assessment as using the resident population can over estimate the risk for areas of low population.

Drowning is significant cause of childhood death in many parts of the globe, and of the half a million people deaths worldwide cause by drowning, 57% of these were among children aged up to 14 years [13,14]. In 26 of the worlds richest nation's injuries were the leading cause of death among children. Drowning was the second leading cause of injury related death, exceeded only by deaths due to road traffic crashes [15]. Drowning is the second lead cause of unintentional injury in children aged 1–14 in the United States, and the leading cause of unintentional injury in children aged 1–3 in every Australian state [3]. The highest annual rates were in the 0–14 age group ranging from 3.1 to 4.2 per 100,000. Each of the age groups identified within the study reported an increase between 2002–3 and 2003–4, when considering the fatality information available it would appear that although fatalities are decreasing in the similar time period, inpatient hospital activity is increasing. It would be interesting to follow those admitted to ascertain their outcomes in terms of secondary treatment of trauma injuries or mortality to calculate the total hospital resource utilisation resulting from water related incidents.

With regard to cause of drownings current global observations assessing how drownings occur in terms of activity prior to the episode, report the following reasons: exposure to environment; participation in water activities; swimming ability; risk taking behaviour; the influence of alcohol and or drugs and vehicle related incidents [16]. The ICD codes do not give predisposing factors prior to the event occurring, nor adequately capture other factors such as the environment or other medical conditions.

This study has reported for the first time on those being admitted to hospital and highlighted the increased magnitude of these events compared to those dying. To avoid duplication and to improve our knowledge of the causal pathways that lead to injury or drowning more work needs to be undertaken linking the two data sets together. Through such work we should be able to provide the evidence to better inform and target our prevention strategies.

## Competing interests

The author(s) declare that they have no competing interests.

## Authors' contributions

HH conceived the paper, undertook the data collection, analysis and literature review. RCW provided the technical expertise on the data, devised the statistical methodology and the provided the interpretation of the results. HH and RCW drafted the paper both contributing equally. Both authors have read and approved the final manuscript.

## Pre-publication history

The pre-publication history for this paper can be accessed here:



## Figures and Tables

**Table 1:** Count and percentage of episodes, occupied bed days and average length of stay by ICD Classification, 1997/8 – 2003/4

*ICD 10 Code*	*Episodes*	*%*	*Bed days*	*%*	*Average length of stay*
V90 Accident to watercraft causing drowning and submersion	56	0.8	102	0.3	1.9
V91 Accident to watercraft causing other injury	247	3.6	1223	3.8	5.3
V92 Water-transport-related drowning/submersion without accident to watercraft	245	3.6	895	2.8	3.8
V93 Accident on board water craft without accident to watercraft not causing drowning or submersion	1100	16.2	4872	15.1	4.6
V94 Other and unspecified water transport accidents	539	7.9	2007	6.2	3.8
W15 Fall from cliff	847	12.5	5644	17.5	7.0
W16 Diving/jumping into water cause injury other than drowning or submersion	1256	18.5	8262	25.6	6.7
W65 Drowning and submersion while in bath-tub	262	3.9	795	2.5	3.2
W66 Drowning and submersion following fall into bath-tub	96	1.4	439	1.4	4.9
W67 Drowning and submersion while in swimming-pool	303	4.5	489	1.5	1.7
W68 Drowning and submersion following fall into swimming-pool	137	2.0	499	1.5	3.8
W69 Drowning and submersion while in natural water	266	3.9	1125	3.5	4.7
W70 Drowning and submersion following fall into natural water	288	4.2	1102	3.4	4.2
W73 Other specified drowning and submersion	154	2.3	653	2.0	4.6
W74 Unspecified drowning and submersion	777	11.4	2731	8.5	3.9
X38 Victim of flood	127	1.9	884	2.7	7.6
X92 Assault by drowning and submersion	66	1.0	239	0.7	5.1
Y21 Drowning and submersion undetermined intent	61	0.9	559	1.7	11.9

**Total**	6793	100.0	32,520	100.0	5.0

**Table 2:** Demography of episodes: Annual episode rates per 100,000 and relative risk (males to females) between 1997/8 and 2003/4

	***1998–9***	***1999/00***	***2000-***	***2001–2***	***2002–3***	***2003–4***
Relative Risk (M:F)	2.7	2.3	2.8	3.0	2.6	2.5
(CIU – CIL)	(2.4 – 3.1)	(2.3 – 3.0)	(2.4 – 3.2)	(2.6 – 3.5)	(2.3 – 3.0)	(2.2 – 2.9)
Overall Rate per 100,000	2.1	2.4	2.0	2.1	2.1	2.2
(CIU – CIL)	(2.0 – 2.4)	(2.1 – 2.7)	(1.8 – 2.3)	(1.8 – 2.4)	(1.8 – 2.3)	(2.0 – 2.6)
						
*Age*						
0–14						
Count	362	395	337	310	292	341
Rate per 100, 000	3.9	4.2	3.6	3.3	3.1	3.6
(CIU – CIL)	(3.5 – 4.3)	(3.8 – 4.6)	(3.2 – 4.0)	(2.9 – 3.7)	(2.8 – 3.5)	(3.2 – 4.0)
						
15–59						
Count	632	726	615	667	640	653
Rate per 100, 000	2.1	2.4	2.0	2.3	2.2	2.2
(CIU – CIL)	(1.9 – 2.3)	(2.3 – 2.6)	(1.9 – 2.2)	(2.0 – 2.4)	(2.0 – 2.3)	(2.0 – 2.4)
						
Over 60						
Count	111	139	126	138	134	170
Rate per 100, 000	1.1	1.4	1.2	1.4	1.3	1.7
(CIU – CIL)	(0.9 – 1.3)	(1.1 – 1.6)	(1.0 – 1.5)	(1.1 – 1.6)	(1.1 – 1.5)	(1.4 – 1.9)

**Table 3:** Count and percentage of episodes by gender and ICD Classification, 1997/8 – 2003/4

	*Males*	*%*	*Females*	*%*
V90 Accident to watercraft causing drowning and submersion	46	81.8	10	18.2
V91 Accident to watercraft causing other injury	195	78.9	52	21.1
V92 Water-transport-related drowning/submersion without accident to watercraft	185	75.5	60	24.5
V93 Accident on board water craft without accident to watercraft not causing drowning or submersion	796	72.4	304	27.6
V94 Other and unspecified water transport accidents	410	76.1	129	23.9
W15 Fall from cliff	697	82.3	150	17.7
W16 Diving/jumping into water cause injury other than drowning or submersion	939	74.8	317	25.2
W65 Drowning and submersion while in bath-tub	141	53.9	121	46.1
W66 Drowning and submersion following fall into bath-tub	55	57.8	41	42.2
W67 Drowning and submersion while in swimming-pool	214	70.5	89	29.5
W68 Drowning and submersion following fall into swimming-pool	98	71.2	39	28.8
W69 Drowning and submersion while in natural water	223	83.7	43	16.3
W70 Drowning and submersion following fall into natural water	208	72.1	80	27.9
W73 Other specified drowning and submersion	118	76.9	36	23.1
W74 Unspecified drowning and submersion	556	71.5	221	28.5
X92 Assault by drowning and submersion	67	53	60	47
X38 Victim of flood	36	54.3	30	45.7
Y21 Drowning and submersion undetermined intent	48	78.7	13	21.3

**Total**	5006	73.7	1787	26.3

**Table 4:** Count and annual episode age specific and all age standardised rate per 100,000 by ICD Code 1997/8 – 2003/4

		Age 0–14		Age 15–59		Age 60+		All ages	
		n	%	n	%	n	%	n	%
V90 Accident to watercraft causing drowning and submersion		7	0.34	42	1.07	7	0.85	56	0.82
	Rate	0.01		0.02		0.01		0.02	
	95%Interval	(0 – 0.04)		(0.01 – 0.04)		(0 – 0.03)		(0.01 – 0.03)	
V91 Accident to watercraft causing other injury		22	1.08	206	5.24	19	2.31	247	3.64
	Rate	0.04		0.12		0.03		0.09	
	95%Interval	(0 – 0.08)		(0.08 – 0.15)		(0 – 0.07)		(0.06 – 0.11)	
V92 Water-transport-related drowning/submersion without accident to watercraft		21	1.03	177	4.50	47	5.71	245	3.61
	Rate	0.04		0.10		0.08		0.08	
	95%Interval	(0 – 0.08)		(0.06 – 0.14)		(0.02 – 0.13)		(0.06- 0.11)	
V93 Accident on board water craft without accident to watercraft not causing drowning or submersion		46	2.26	821	20.87	233	28.31	1100	16.19
	Rate	0.08		0.46		0.38		0.37	
	95%Interval	(0.02 – 0.14)		(0.38 – 0.54)		(0.26 – 0.5)		(0.31 – 0.42)	
V94 Other and unspecified water transport accidents		27	1.33	468	11.90	44	5.35	539	7.93
	Rate	0.05		0.26		0.07		0.19	
	95%Interval	(0 – 0.09)		(0.2 – 0.32)		(0.02 – 0.12)		(015 – 0.22)	
W15 Fall from cliff		160	7.85	627	15.94	60	7.29	847	12.47
	Rate	0.29		0.35		0.10		0.30	
	95%Interval	(0.18 – 0.4)		(0.28 – 0.42)		(0.04 – 0.16)		(0.25 – 0.35)	
W16 Diving/jumping into water cause injury other than drowning or submersion		331	16.25	844	21.46	81	9.84	1256	18.49
	Rate	0.59		0.47		0.13		0.45	
	95%Interval	(0.44 – 0.75)		(0.4 – 0.55)		(0.06 – 0.2)		(0.39 – 0.51)	
W65 Drowning and submersion while in bath-tub		209	10.26	28	0.71	25	3.04	262	3.86
	Rate	0.38		0.02		0.04		0.10	
	95%Interval	(0.25 – 0.5)		(0 – 0.03)		(0 – 0.08)		(0.07 – 0.13)	
W66 Drowning and submersion following fall into bath-tub		54	2.65	12	0.31	30	3.65	96	1.41
	Rate	0.10		0.01		0.05		0.03	
	95%Interval	(0.03 – 0.16)		(0 – 0.02)		(0.01 – 0.09)		(0.02- 0.05)	
W67 Drowning and submersion while in swimming-pool		225	11.05	67	1.70	11	1.34	303	4.46
	Rate	0.40		0.04		0.02		0.12	
	95%Interval	(0.27 – 0.53)		(0.02 – 0.06)		(0 – 0.04)		(0.9 – 0.15)	
W68 Drowning and submersion following fall into swimming-pool		105	5.15	24	0.61	8	0.97	137	2.02
	Rate	0.19		0.01		0.01		0.05	
	95%Interval	(0.1 – 0.28)		(0 – 0.03)		(0 – 0.04)		(0.03 – 0.07)	
W69 Drowning and submersion while in natural water		82	4.03	146	3.71	38	4.62	266	3.92
	Rate	0.15		0.08		0.06		0.09	
	95%Interval	(0.07 – 0.23)		(0.05 – 0.11)		(0.01 – 0.11)		(0.07 – 0.12)	
W70 Drowning and submersion following fall into natural water		133	6.53	101	2.57	54	6.56	288	4.24
	Rate	0.24		0.06		0.09		0.10	
	95%Interval	(0.14 – 0.34)		(0.03 – 0.08)		(0.03 – 0.15)		(0.07 – 0.13)	
W73 Other specified drowning and submersion		118	5.79	28	0.71	8	0.97	154	2.27
	Rate	0.21		0.02		0.01		0.06	
	95%Interval	(0.12 – 0.31)		(0 – 0.03)		(0 – 0.04)		(0.04 – 0.08)	
W74 Unspecified drowning and submersion		402	19.73	279	7.09	96	11.66	777	11.44
	Rate	0.72		0.16		0.16		0.28	
	95%Interval	(0.55 – 0.9)		(0.11 – 0.2)		(0.08 – 0.23)		(0.23 – 0.33)	
X38 Victim of flood		71	3.49	16	0.41	40	4.86	127	1.87
	Rate	0.13		0.01		0.07		0.04	
	95%Interval	(0.05 – 0.2)		(0 – 0.02)		(0.02 – 0.11)		(0.03 – 0.06)	
X92 Assault by drowning and submersion		9	0.44	23	0.58	34	4.13	66	0.97
	Rate	0.02		0.01		0.06		0.02	
	95%Interval	(0 – 0.04)		(0 – 0.03)		(0.01 – 0.1)		(0.01 – 0.03)	
Y21 Drowning and submersion undetermined intent		15	0.74	24	0.61	22	2.67	61	0.90
	Rate	0.03		0.01		0.04		0.02	
	95%Interval	(0 – 0.06)		(0 – 0.03)		(0 – 0.07)		(0.01 – 0.03)	

Total		2037	100.0	3933	100.0	823	100.0	6793	100.0
	Rate	3.66		2.21		1.34		2.39	
	95%Interval	(3.27 – 4.05)		(2.04 – 2.38)		(1.12 – 1.57)		(2.25 – 2.53)	
